# Dyslexia screening? As easy as a game. The Starlex project: evidence from an Italian pilot study

**DOI:** 10.3389/fpsyg.2026.1872307

**Published:** 2026-09-25

**Authors:** Alessandra Micalizzi, Sara Zaccaria

**Affiliations:** 1 SAE Institute of Milan, Milan, Italy; 2 Hogrefe Editore, Florence, Italy

**Keywords:** dyslexia, screening, self-efficacy, serious game, videogame studies

## Abstract

In recent years, serious games have gained increasing attention as effective tools for supporting learning and assessing emerging cognitive skills in early educational settings. Within this perspective, the present study introduces Starlex, a serious game designed to screen pre-literacy skills in children aged 5–7 years, a critical stage for the development of foundational reading and writing abilities. Developed within a PNRR-funded project, the game comprises four mini-games aimed at assessing key predictors of reading acquisition, including visual discrimination and working memory. Following an initial phase of playability and usability testing, Starlex was administered to a pilot sample of 84 children in early education contexts. Game-based performance was compared with standardized assessments targeting the same cognitive domains. Findings indicate that Starlex provides a promising and engaging approach to the early identification of learning-related difficulties, showing good alignment with traditional measures, as well as satisfactory levels of usability and acceptance among young learners. While further refinements are needed to enhance the robustness of the tool, its replicability emerges as a key feature, supporting sustained engagement and enabling continuous observation of children’s performance over time. Overall, this study highlights the potential of serious games as complementary tools for formative assessment and early screening within educational environments, contributing to more accessible and child-friendly evaluation practices.

## 1 Serious games: definition, benefits, and relevance for children

In contemporary debates, play is understood as a complex and generative process that supports learning, adaptation, and identity construction across the lifespan (Winnicott, 1991; Mead, 1967). Rather than being merely recreational, play provides a protected space for experimentation, in which individuals can explore roles, strategies, and emotional responses within a framework that legitimizes error and encourages exploration. From this perspective, applied and serious games do not introduce a new function but rather make explicit a dimension already inherent to play.

Serious games are defined as environments designed for purposes beyond entertainment, including educational, training, or clinical goals (Apt, 1970; Laamarti et al., 2014; Mayer, 2019; Hornos-Arias et al., 2025). They are characterized by the coherent integration of learning objectives, rules, feedback, and interactive scenarios (Plass et al., 2015; Plass et al., 2020). A key theoretical perspective conceptualizes serious games as protected spaces of exploration, comparable to a digital “magic circle,” in which children can engage in tasks, decision-making, and trial-and-error processes without the immediate negative consequences typical of real-world contexts. Evidence consistently describes these environments as controlled, immersive, and “risk-free,” enabling the development of decision-making, adaptation, and problem-solving skills without the pressure associated with traditional assessment settings (Castillo-Parra et al., 2022; Steinkuehler and Duncan, 2008). In particular, the use of simulated and branched scenarios allows players to act, fail, receive feedback, and try again, transforming error from a punitive element into a core component of the learning process (López-Serrano et al., 2025). This is especially relevant in early childhood and in contexts of learning difficulties, where it helps reduce performance pressure and reframes tasks as exploratory experiences.

Alongside this dimension, motivation and engagement play a central role. Digital game environments support attention, participation, and persistence over time (Prensky, 2003), also by activating fundamental psychological needs such as competence, autonomy, and relatedness (Ryan and Deci, 2000). Mechanisms such as challenge, progression, immediate feedback, and trial-and-error foster active and sustained learning (Sandí-Delgado et al., 2022; Klock and Dick, 2021; Gee, 2003; Delgado and Mendes, 2023; Lampropoulos and Sidiropoulos, 2024), although motivational outcomes are not always consistent and may depend on contextual factors and familiarity with digital media (Polydoros and Antoniou, 2025). From a design perspective, the effectiveness of serious games depends on the ability to balance affective, motivational, cognitive, and socio-cultural dimensions (Plass et al., 2015; Plass et al., 2020), avoiding both overstimulation and the reduction of gameplay to a disguised exercise (Barz et al., 2024). Research also shows that specific design choices produce differentiated effects: repetition supports skill consolidation (Clark et al., 2016), competition is not necessary for engagement (Cagiltay et al., 2015), and three-dimensional environments may increase activation but also cognitive load (Lamb et al., 2018; Wouters and Van Oostendorp, 2013). At the same time, gender differences highlight the potential of games as tools for empowerment when designed inclusively (Vogel et al., 2006).

Despite these opportunities, the literature also identifies relevant challenges. These include the risk of compromising effectiveness by prioritizing educational goals at the expense of the ludic dimension (Barz et al., 2024), the possibility of simplified or stereotypical representations of complex issues (Chess, 2020; Nardone, 2023), and ethical concerns related to data management and the vulnerability of users. Another key conceptual dimension concerns agency: in serious games, the player is positioned at the center of the experience and learns through action, choice, and experimentation. Interactivity, adaptive pacing, and immediate feedback support active and self-directed learning processes (Polydoros and Antoniou, 2025; López-Serrano et al., 2025; Spangenberger et al., 2025), aligning with generative pedagogical models in which meaning is constructed through doing.

This dimension is closely connected to the role of games as spaces for inclusion and social interaction. Serious games can foster collaboration, social learning, and the development of transversal skills (Zheng et al., 2021; López-Serrano et al., 2025), helping to reduce stigma and support more accessible educational contexts, particularly for children with specific learning difficulties (Ekawati et al., 2024). When designed with attention to usability, simplicity, and accessibility, they offer immersive and adaptable environments capable of responding to diverse needs and learning paces (Hornos-Arias et al., 2025). In this sense, serious games can be understood not only as tools for enhancing engagement, but as educational contexts in which protection, agency, and social interaction intersect, allowing children to experience learning as meaningful, shared, and non-stigmatizing.

## 2 Serious games in dyslexia screening: applications, rationale, strengths, and limitations

In recent years, the use of serious games in the field of specific learning disorders has progressively expanded from intervention to assessment and early screening, although the latter remains less developed and systematized. Available evidence suggests that gamified digital environments can be used to ecologically assess a range of cognitive and pre-academic skills relevant to reading, such as visuospatial attention, working memory, and phonological abilities—key domains implicated in dyslexia (Bertoni et al., 2021; Franceschini et al., 2015). In particular, studies on action video games indicate that interaction with game environments can influence attentional processes and phonological decoding, suggesting their potential not only for training but also for identifying cognitive patterns associated with early risk (Bertoni et al., 2024).

More broadly, this application scenario aligns with the increasing use of digital tools for learning disorder screening, aimed at overcoming some limitations of traditional assessments through automated data collection, scalability, and opportunities for longitudinal monitoring. Serious games enable the integration of cognitive tasks into interactive environments, allowing continuous and multimodal data collection on children’s behavior (Hornos-Arias et al., 2025). In this sense, gameplay becomes a form of implicit assessment, where performance is embedded within the activity rather than separated from it.

The widespread familiarity of children with digital devices, together with the possibility of designing highly intuitive interfaces, makes game-based assessment suitable even for very young children, including those in preschool. Several studies illustrate this potential. DIESEL-X (Geurts et al., 2015) is a video game developed for preschool screening, including three mini-games targeting predictive indicators of dyslexia: frequency modulation detection, phonological awareness (through identification of rhyming words), and letter knowledge. Rauschenberger et al. (2025) developed a universal game-based screening tool grounded in the analysis of errors made by individuals with dyslexia across languages, incorporating parameters such as auditory and visual perception; their study involved 313 children (116 with dyslexia) and employed machine learning models trained on gameplay data. More recent developments include augmented reality applications, such as RATSEL, a game-based assessment tool designed to enhance school evaluation practices by providing a more engaging and motivating environment for children with dyslexia (Sorna Shanthi et al., 2022). Similarly, Dytective (Rello et al., 2016) is a game designed to identify dyslexia risk in English and Spanish through linguistic tasks based on common error patterns, showing the ability to distinguish between children with and without dyslexia across languages.

The rationale for using serious games in screening is closely linked to their intrinsic characteristics. First, they can increase engagement and reduce resistance to assessment by framing evaluation as a less threatening activity. Game environments have been shown to support motivation, active participation, and persistence, even in the presence of difficulties (Ekawati et al., 2024). Second, serious games allow for adaptive and personalized experiences: difficulty levels can be dynamically adjusted, feedback can be provided in real time, and tasks can be tailored to individual user profiles, enabling more fine-grained assessment of strengths and weaknesses (Hornos-Arias et al., 2025). This is particularly relevant in developmental disorders, which are characterized by high variability and require sensitive and flexible assessment tools. In addition, the integration of assessment and intervention within the same environment represents a further advantage, as serious games can simultaneously function as diagnostic tools and training contexts.

However, several critical issues remain. First, robust clinical validation is still lacking, limiting the use of serious games as formal diagnostic tools; they are more commonly employed as complementary instruments rather than replacements for standardized assessment batteries. Second, performance in game-based environments may be influenced by extraneous variables such as familiarity with video games, digital skills, and individual preferences. As highlighted by Polydoros and Antoniou (2025), the effects of serious games on motivation and performance are not uniform and may vary depending on individual and contextual factors. Consequently, observed performance may not exclusively reflect target cognitive abilities but also non-cognitive influences.

Finally, a key challenge concerns the design of serious games for screening purposes. To ensure validity, such tools must be grounded in solid theoretical models of the abilities being assessed and adhere to rigorous psychometric criteria. However, it remains unclear which specific game design elements (e.g., feedback type, reward systems, level of complexity) are most effective for assessing different competencies (Hornos-Arias et al., 2025).

## 3 Starlex: a serious game for dyslexia screening

The Starlex project was developed in response to the need to overcome the limitations of traditional tools for the early identification of learning disorders and to propose assessment approaches that combine scientific rigor with attention to children’s emotional experience. Targeted at children aged 6–7 years, Starlex is a digital game designed to detect early indicators of dyslexia risk without making its evaluative function explicit. This approach reduces anxiety and performance pressure by preventing children from perceiving the activity as a test. The design is grounded in a game-first approach, in which the game is not a support for measurement but the primary context in which observation takes place (Gee, 2003; Petri et al., 2024).

From a design perspective, Starlex integrates principles from applied games, game-based assessment, and child-centered interaction design. Unlike many serious games, where gameplay is subordinated to explicit learning or diagnostic goals, the evaluative dimension is embedded within the game mechanics themselves, which are designed to be intuitive, exploratory, and intrinsically motivating. As a result, children are not required to demonstrate skills directly; instead, relevant data emerge naturally through their interaction with the ludic environment.

The game architecture consists of a sequence of short, independent activities, combining narrative elements, visual feedback, and simple interactions. These activities are designed to target specific predictors of dyslexia risk, such as visual discrimination and working memory. Game mechanics are structured to elicit observable behavioral indicators—including response times, accuracy, and interaction patterns—without making the assessment process explicit to the child (Zaccaria, 2022).

Accessibility represents a central design principle. Control systems are kept simple and consistent across activities to prevent technical difficulties from interfering with performance. Similarly, audiovisual elements are designed to sustain attention without generating cognitive overload. Visual design and feedback systems avoid strong distinctions between success and failure, instead favoring neutral or encouraging responses that support exploration and maintain engagement without emphasizing competition.

During gameplay, performance data are continuously collected and made available through an interface designed for adult users. Although the project is still under development, its framework is based on a stealth assessment model: data are captured unobtrusively and processed into performance indicators derived from gameplay behavior. These indicators do not have formal diagnostic value but can provide preliminary performance indicators associated with cognitive domains relevant to dyslexia screening. In this sense, Starlex is conceived as a supportive and orienting tool that complements, rather than replaces, clinical or educational assessment, providing useful insights even in contexts where no formal diagnostic process has yet been initiated.

### 3.1 Game description

The project is centered around Sky, an extraterrestrial character who lands on an unknown planet, Soricea. Here, Sky is tasked with exploring the environment, interacting with its inhabitants, and contributing to the construction of a new colony. To achieve this, the player must collect resources and complete a series of tasks. Only after accomplishing these activities and leaving a message of gratitude to the planet’s inhabitants can Sky begin the return journey, which ultimately requires completing a code necessary for the spacecraft’s departure.

The gameplay experience unfolds across four main environments, each accompanied by brief texts and dialogues that structure the interaction between Sky and the inhabitants of Soricea, providing a coherent and engaging narrative framework.

The core activities are implemented through four mini games inspired by familiar gameplay mechanics, adapted to align with the project’s assessment goals. In the first task, a variant of a “whack-a-mole” game, players are required to select only the moles displaying a specific symbol corresponding to that shown on a fruit crate, targeting visual discrimination and recognition skills. The second mini game, inspired by snake, involves guiding a small spacecraft to collect stones marked with a target symbol while avoiding distractors and obstacles, thus engaging visual analysis and visuospatial orientation.

The third mini game, set in a domestic context, requires players to correctly deliver letters by matching the name on an intercom with the name on an envelope; this task targets the visual discrimination of letter strings (non-words). Finally, in the concluding phase, players access the spacecraft and must complete a sequence of symbol-matching tasks, like a memory game, engaging working memory on visual stimuli.

At this stage of development, the game focuses primarily on visual discrimination and working memory. Although the present pilot focuses on visual discrimination and working memory, dyslexia risk is widely understood as a multidimensional construct involving phonological awareness, rapid automatized naming, and auditory processing. Future versions of Starlex will integrate these predictors to improve construct coverage and increase the ecological validity of the screening model. The overall design follows an inclusive perspective, with attention to intersectionality. Characters are deliberately free from explicit gender markers, and the science-fiction setting emphasizes themes of encounter, discovery, and cultural exchange, avoiding narratives centered on conquest or domination.

### 3.2 Validation phase: usability and screening

The study was structured into two complementary phases: an initial exploratory phase focused on usability, followed by a more structured phase aimed at the preliminary analysis of performance data, including aspects of validity and reliability. The exploratory phase, conducted in July 2025, involved a sample of 20 children aged 5–7 years. Its primary objective was to assess the functionality of the game mechanics and the level of engagement generated by the experience. This phase allowed the identification of design issues, including technical bugs, and supported the calibration of task difficulty, particularly in terms of speed and responsiveness requirements.

The data collection phase involved a larger sample of 104 children attending the first and second grades of primary school at the Istituto Gonzaga in Milan, in collaboration with the school’s inclusion coordinator. Twenty cases were excluded due to incomplete gameplay sessions, technical interruptions, or inconsistent data registration, resulting in a final sample of 84 participants. The aim was to evaluate the system’s ability to generate valid and reliable performance indicators comparable to those obtained through traditional early screening protocols for dyslexia, while preserving the centrality of the playful and emotional experience (Zaccaria, 2022). To this end, selected tasks from the PRCR-2/2009 battery (Cornoldi et al., 2009) were administered to assess visual analysis and discrimination skills, both sequential and simultaneous, which are widely recognized as key precursors of reading and writing acquisition.

The study adopted an exploratory-comparative design. Participants first completed traditional paper-and-pencil tasks and subsequently interacted with the game on tablets in familiar environments, to minimize situational stress. Gameplay sessions were not time-constrained and did not include performance-oriented instructions, in line with the principles of game-based and stealth assessment, which emphasize intrinsic motivation and ecological validity (Shute et al., 2016; Shute and Ventura, 2013).

For Game 1, the system recorded the total number of responses, correct responses, omissions (failure to select a target stimulus), and errors (selection of a non-target stimulus). A total correct score was computed as: total responses − omissions − errors. For Game 2, only the number of target elements successfully collected was recorded. For Game 3, the number of correct responses required to complete the task was recorded. For Game 4, the number of attempts required to complete the memory sequence was used as the performance indicator. No additional scoring algorithms were applied.

Children completed the activities under naturalistic classroom conditions. No randomization procedures were implemented, and the order of game presentation was not experimentally manipulated. Information regarding participants’ previous gaming experience or familiarity with digital devices was not collected and therefore could not be controlled statistically. These data were subsequently processed to derive indicators related to specific cognitive skills, particularly visual discrimination and working memory, both widely recognized as critical in dyslexia research (Petri et al., 2024). Importantly, children experienced the activity solely as gameplay, without awareness of its evaluative dimension, consistent with the principles of stealth assessment (Micalizzi, 2022).

Each session lasted approximately 20–25 min. Children completed the paper-based tasks individually in a quiet classroom setting and subsequently played Starlex on 10-inch touchscreen tablets under teacher supervision. The order of administration was kept constant across participants.

## 4 Analysis of quantitative and performance indicators

The pilot sample, after excluding invalid cases, consisted of 84 children aged 6–7 years (M = 6.53 ± 0.50), of whom 64% were male.

The following quantitative indicators were collected and analyzed across the four games:

- Game 1 (whack-a-mole-like task): total responses, correct responses, omissions, and errors. A total correct score was computed by subtracting omissions and errors from the total number of responses.
- Game 2 (snake-like task): number of target elements collected.
- Game 3 (letter-matching task): number of correct responses.
- Game 4 (memory-like task): number of attempts required to complete the code.

For Game 1, most responses were correct, while omissions were approximately twice as frequent as errors. Apart from omissions, variables did not follow a normal distribution, although the total correct score showed only a slight deviation from normality due to kurtosis. Scores in Game 2 were normally distributed; however, the task appeared relatively challenging for the target age group, with the observed mean closer to the theoretical minimum than to the midpoint. Only 52.3% of children successfully completed Game 3, requiring on average nearly five responses to reach the correct solution; the distribution of responses was approximately normal. In Game 4, 69 children (80.2% of the sample) successfully completed the task, requiring an average of 16 attempts; this variable did not show a normal distribution (Table 1).

**Table 1:** Descriptive statistics for game performance measures.

Game	Measure	M	DS	Min	Max	Skewness	Kurtosis
Game 1	Total responses	28.5	10.78	4	65	1	1.75
	Correct responses	17.89	10.92	−4	60	1.2	2.44
	Errors	3.44	5.22	0	39	4.38	25.76
	Omissions	7.2	1.65	4	12	0.19	0.02
	Total correct score	7.24	14.04	−43	55	0.16	2.62
Game 2	Correct responses	0	5	1.53	1.24	0.96	0.17
Game 3	Correct responses	4.81	1.32	2	7.5	−0.06	−0.68
Game 4	Number of attempts	16	7.03	8	63	4.58	29.4

To examine the validity of the games, multiple dimensions were considered: relationships among game scores, associations with socio-demographic variables (age and gender), and correlations with established psychological measures.

Descriptive statistics were computed for all variables. Pearson correlations were used to examine associations between game-based and standardized measures. Independent-samples *t*-tests were conducted to compare performance across age, gender, and normative/difficulty subgroups. Chi-square analyses were used for categorical completion variables. Effect sizes were calculated using Cohen’s *d*. Given the exploratory nature of the study and the non-normal distribution of some variables, results should be interpreted cautiously.

### 4.1 Relationships among game scores

Correlation analyses for Game 1 revealed statistically significant associations in the expected direction. Positive correlations were found between correct responses and total correct score (*r* = 0.84, *p* < 0.001), and between errors and omissions (*r* = 0.46, *p* < 0.001). Negative correlations emerged between accuracy-related scores and error-related measures (*r* = −0.43, *p* < 0.001, omissions and correct responses; *r* = −0.59, *p* < 0.001, total correct score and errors; *r* = −0.62, *p* < 0.001, total correct score and omissions). No statistically significant correlations were found between scores from different games. This aspect should be interpreted with caution. The four mini-games were designed to assess partially distinct cognitive processes rather than different manifestations of a single latent construct. Consequently, strong inter-game correlations were not necessarily expected. Nevertheless, future studies should further investigate the relationships among these measures and their contribution to a broader multidimensional model of dyslexia-risk screening.

Given that correlations across games were calculated only for children who successfully completed Games 3 and 4, further analyses were conducted to examine whether task completion was associated with differences in performance in other games. Results showed that children who reached the end of Game 4 achieved significantly higher scores in both total responses and correct responses in Game 1, with moderate effect sizes. Completion status should not be interpreted as a pass/fail outcome, but merely as an indicator of whether the full task sequence was completed during gameplay.

No significant differences were observed for the other Game 1 indicators. This group also showed significantly better performance in Game 2, with a moderate Cohen’s *d* (see Table 2).

**Table 2:** Differences in scores between children who completed and did not complete Game 4.

Game	Measures	Completed M	Completed DS	Not completed M	Not completed DS	*t*	df	*p*	*d* Cohen
Game 1	Total responses	29.79	11	23.24	8.19	2.3	84	<0.05	0.50
	Correct responses	19.23	11.21	12.44	7.8	2.36	84	<0.05	0.51
	Errors	3.48	5.55	3.28	3.7	0.14	84	0.89	0.03
	Omissions	7.12	1.71	7.53	1.39	−0.91	84	0.37	0.20
	Total correct score	8.63	14.63	1.63	9.77	1.87	84	0.07	0.41
Game 2	Correct responses	1.67	1.25	0.94	1.02	2.23	84	<0.50	0.49
Game 3	Correct responses	4.78	1.26	4.94	1.66	−0.31	43	0.76	0.09

*d* ≤ 0.20: small, 0.21 ≤ *d* ≤ 0.50: moderate, 0.51 ≤ *d* ≤ 0.80: medium, *d* ≥ 0.81: high.

No statistically significant differences were observed in performance on the other games between subgroups of children who did and did not complete Game 3.

### 4.2 Relationships with socio-demographic variables

With regard to gender (see Table 3), and in line with expectations, no statistically significant differences were found in mean scores between boys and girls across all game indicators.

**Table 3:** Gender differences.

Game	Measures	Male M	Male DS	Female M	Female DS	*t*	df	*p*	*d* Cohen
Game 1	Total responses	29.25	12.08	27.16	8.01	0.86	84	0.39	0.19
	Correct responses	17.82	12.31	18.02	8.08	−0.08	84	0.94	0.02
	Errors	4.2	6.26	2.1	1.9	1.81	84	0.07	0.39
	Omissions	7.11	1.64	7.37	1.67	−0.71	84	0.48	0.15
	Total correct score	6.51	16.23	8.54	9.01	−0.64	84	0.52	0.14
Game 2	Correct responses	1.57	1.25	1.45	1.24	0.43	84	0.67	0.09
Game 3	Correct responses	4.94	1.35	4.53	1.26	0.98	43	0.33	0.30
Game 4	Number of attempts	16.83	8.47	14.7	3.63	1.23	67	0.22	0.30

*d* ≤ 0.20: small, 0.21 ≤ *d* ≤ 0.50: moderate, 0.51 ≤ *d* ≤ 0.80: medium, *d* ≥ 0.81: high.

To further examine the association between gender and task completion in Games 3 and 4, chi-square tests were conducted, with gender as the independent variable and task completion as the dependent variable. Results were non-significant in both cases (Game 3: χ^2^(1) = 0.30, *p* = 0.58; Game 4: χ^2^(1) = 1.44, *p* = 0.23).

In contrast, age was expected to influence performance, with older children showing better outcomes than younger ones. Statistically significant differences were observed in the expected direction for Game 1, specifically in the number of responses, number of correct responses, and total performance score, with medium effect sizes. A similar pattern was found for Game 2 (see Table 4).

**Table 4:** Age differences.

Game	Measures	6y M	6y DS	7y M	7y DS	*t*	df	*p*	*d* Cohen
Game 1	Total responses	24.7	8.83	31.79	1.32	−3.2	84	<0.01	0.70
	Correct responses	14.04	7.24	21.24	12.46	−3.21	84	<0.01	0.70
	Errors	3.56	6.41	3.34	3.97	0.2	84	0.84	0.04
	Omissions	7.39	1.74	7.04	1.56	0.97	84	0.34	0.21
	Total correct score	3.09	11.75	10.86	14.96	−2.65	84	<0.05	0.58
Game 2	Correct responses	1.16	0.87	1.84	1.42	−2.65	84	<0.05	0.58
Game 3	Correct responses	4.58	1.33	4.99	1.31	1.06	43	0.30	0.32
Game 4	Number of attempts	16.38	10.24	15.8	4.62	0.32	67	0.75	0.08

*d* ≤ 0.20: small, 0.21 ≤ *d* ≤ 0.50: moderate, 0.51 ≤ *d* ≤ 0.80: medium, *d* ≥ 0.81: high.

Similarly, to the analyses conducted for age-related performance differences, chi-square tests were computed to examine the association between age and task completion in Games 3 and 4, given the absence of significant differences in the number of attempts and responses in these tasks. Results indicate that successful completion of Game 4 was significantly associated with age (χ^2^(1) = 19.30, *p* < 0.001), whereas no such association was observed for Game 3 (χ^2^(1) = 0.16, *p* = 0.69).

### 4.3 Relationships with other measures

To further assess the validity of the measures obtained through Starlex, three paper-and-pencil tasks were administered to the same group of children. These tasks, drawn from a validated battery assessing visual prerequisites of reading and writing (PRCR-2/2009; Cornoldi et al., 2009), targeted the same underlying constructs:

- *Letter recognition*: identifying target letters among four alternatives with graphical or spatial alterations.
- *Two-letter search*: identifying two target letters within an ordered configuration.
- *Letter sequence search*: identifying a sequence of three letters within strings of closely spaced letters.

Based on performance in these tasks, the sample was divided into two groups: children within the normative range and children showing difficulties.

Correlation analyses indicate adequate convergent validity for Games 1 and 2 with the paper-based screening tasks (see Table 5). The number of responses in Game 1 showed statistically significant, moderate correlations in the expected direction with the *Letter search task* (omissions) and the *Letter sequence search task* (correct responses and number of errors). A similar pattern was observed for the number of correct responses and the total correct score in Game 1, with higher correlation coefficients, particularly with omissions in the *Letter search task*.

**Table 5:** Correlations with validation measures.

Game	Measures	Two-letter search omissions	Letter sequence search correct responses	Letter sequence search errors	Letter recognition errors
Game 1	Total responses	−0.26*	0.23*	−0.25*	−0.04
	Correct responses	−0.42***	0.30**	−0.31**	−0.15
	Errors	0.27*	−0.15	0.13	0.13
	Omissions	0.25*	−0.16	0.15	0.27*
	Total correct score	−0.46***	0.30**	−0.31**	−0.20
Game 2	Correct responses	−0.26*	0.33**	−0.33**	−0.08
Game 3	Correct responses	0.13	0.04	−0.04	−0.12
Game 4	Number of attempts	−0.03	−0.05	0.03	0.01

*** *p* < 0.0011; ** *p* < 0.01; * *p* < 0.05.

Game 2 scores also showed statistically significant moderate correlations, in the expected direction, with both the *Letter search* and *Letter sequence search tasks*. In contrast, no significant correlations were found between the paper-based measures and the scores of Games 3 and 4. This finding may reflect a partial mismatch between the constructs assessed by the validation battery and those targeted by Games 3 and 4. Whereas the PRCR tasks primarily focus on visual discrimination and visual search abilities, Games 3 and 4 were designed to engage additional processes, including visual matching of letter strings and working memory. Future studies should therefore employ validation measures more closely aligned with these specific cognitive domains.

To further examine the validity of Games 3 and 4, differences in performance on the validation tasks were analyzed based on whether children successfully completed each game.

Results indicate that omissions in the *Letter search task* were significantly lower, with a medium effect size, in the subgroup of children who did not complete Game 4 compared to those who successfully completed it (see Table 6). No statistically significant differences were observed for Game 3 across the same validation measures.

**Table 6:** Differences in validation task scores between children who did and did not complete Game 3.

Measure	Completed M	Completed DS	Not completed M	Not completed DS	*t*	df	*p*	*d* Cohen
Two-letter search (omissions)	8.41	5.98	13.24	10.52	−2.52	84	<0.05	0.55
Letter sequence search (correct responses)	17.84	7.68	15.24	7.68	1.25	84	0.21	0.27
Letter sequence search (errors)	15.62	7.91	18.47	7.6	−1.34	84	0.18	0.29
Letter recognition (errors)	2.03	3.44	3.82	4.14	−1.85	84	0.07	0.40

*d* ≤ 0.20: small, 0.21 ≤ *d* ≤ 0.50: moderate, 0.51 ≤ *d* ≤ 0.80: medium, *d* ≥ 0.81: high.

Given that the three validation tasks allow for distinguishing between children within the normative range and those experiencing difficulties, further analyses examined differences in game performance between these subgroups. This provides an estimate of the discriminant validity of the games, that is, their ability to differentiate between typically developing children and those with learning difficulties.

Findings show that children within the normative range on the *Letter search task* achieved significantly higher performance in Game 1 compared to children in the difficulty range, across all game indicators. The largest effects were observed for the number of correct responses and the total correct score, both showing large effect sizes (see Table 7).

**Table 7:** Differences in game scores based on performance level in the *Letter search task*.

Game	Measures	Normative range M	Normative range DS	Difficulties M	Difficulties DS	*t*	df	*p*	*d* Cohen
Game 1	Total responses	31.8	11.92	26.44	9.56	2.3	84	<0.05	0.50
	Correct responses	23.32	12.6	14.51	8.18	3.93	84	<0.001	0.86
	Errors	2.02	2.08	4.33	6.3	−2.04	84	<0.05	0.45
	Omissions	6.53	1.27	7.62	1.72	−3.14	84	<0.01	0.69
	Total correct score	14.77	13.73	2.56	12.15	4.31	84	<0.001	0.94
Game 2	Correct responses	1.73	1.24	1.4	1.23	1.23	84	0.22	0.27
Game 3	Correct responses	4.68	1.72	4.88	1.58	−0.54	84	0.59	0.12
Game 4	Number of attempts	16.52	12.83	17.15	12.03	−0.23	84	0.82	0.05

*d* ≤ 0.20: small, 0.21 ≤ *d* ≤ 0.50: moderate, 0.51 ≤ *d* ≤ 0.80: medium, *d* ≥ 0.81: high.

For Games 3 and 4, chi-square analyses were conducted with performance level in the *Letter search task* as the independent variable and task completion as the dependent variable. In both cases, results were not statistically significant (Game 4: χ^2^(1) = 0.09, *p* = 0.77; Game 3: χ^2^(1) = 0.01, *p* = 0.91).

The division of the sample into two subgroups (normative vs. difficulty range) based on performance in the *Letter sequence search task* revealed statistically significant differences in the expected direction for Game 1. Specifically, differences emerged in the number of correct responses and the total correct score, both showing medium effect sizes (see Table 8).

**Table 8:** Differences in game scores based on performance level in the *Letter sequence search task*.

Game	Measures	Normative range M	Normative range DS	Difficulties M	Difficulties DS	*t*	df	*p*	*d* Cohen
Game 1	Total responses	29.8	10.8	25.13	10.21	1.82	84	0.07	0.40
	Correct responses	19.57	11.31	13.54	8.62	2.36	84	<0.05	0.51
	Errors	3.13	3.88	4.26	7.73	−0.91	84	0.37	0.20
	Omissions	7.04	1.67	7.63	1.55	−1.49	84	0.14	0.33
	Total correct score	9.41	13.57	1.65	13.95	2.36	84	<0.05	0.51
Game 2	Correct responses	1.67	1.38	1.16	0.65	1.72	84	0.09	0.38
Game 3	Correct responses	4.71	1.72	5.03	1.38	−0.82	84	0.42	0.18
Game 4	Number of attempts	16.11	12.6	18.96	11.36	−0.96	84	0.34	0.21

*d* ≤ 0.20: small, 0.21 ≤ *d* ≤ 0.50: moderate, 0.51 ≤ *d* ≤ 0.80: medium, *d* ≥ 0.81: high.

Consistent with previous findings, no statistically significant associations were found between performance level in the *Letter sequence search task* and completion of Games 3 and 4 (Game 3: χ^2^(1) = 1.38, *p* = 0.24; Game 4: χ^2^(1) = 0.58, *p* = 0.45).

When examining game performance in relation to performance levels in the *Letter recognition task*, results showed statistically significant differences in the expected direction, with medium to moderate effect sizes. These differences were observed for correct responses, omissions, and total correct score in Game 1 (see Table 9).

**Table 9:** Differences in game scores based on performance level in the *Letter recognition task*.

Game	Measures	Normative range M	Normative range DS	Difficulties M	Difficulties DS	*t*	df	*p*	*d* Cohen
Game 1	Total responses	29.49	11.57	27.18	9.64	0.98	84	0.33	0.22
	Correct responses	19.9	12.61	15.23	7.55	2	84	<0.05	0.44
	Errors	2.8	3.49	4.29	6.83	−1.32	84	0.19	0.29
	Omissions	6.86	1.52	7.66	1.72	−2.3	84	<0.05	0.50
	Total correct score	10.24	14.86	3.27	11.93	2.34	84	<0.05	0.51
Game 2	Correct responses	1.67	1.34	1.34	1.08	1.23	84	0.22	0.27
Game 3	Correct responses	4.86	1.87	4.72	1.26	0.39	84	0.70	0.09
Game 4	Number of attempts	16.22	12.28	17.81	12.37	−0.59	84	0.56	0.13

*d* ≤ 0.20: piccolo, 0.21 ≤ *d* ≤ 0.50: moderato, 0.51 ≤ *d* ≤ 0.80: medio, *d* ≥ 0.81: elevato.

Chi-square analyses conducted to examine the association between performance level in the *Letter recognition task* and completion of Games 3 and 4 were not statistically significant (Game 3: χ^2^(1) = 2.52, *p* = 0.11; Game 4: χ^2^(1) = 0.85, *p* = 0.36).

Overall, the results of the pilot study indicate that Starlex can capture quantitative behavioral indicators through gameplay that are meaningfully related to visual prerequisites of reading and writing identified in the literature. Among the four tasks, Game 1 appears to be the most robust in terms of design and performance, showing stronger evidence of both convergent and discriminant validity compared to the other games.

### 4.4 Toward a qualitative interpretation of the testing experience

The administration phase also provided valuable qualitative insights into the gameplay experience, supporting a more nuanced interpretation of the results and informing future system development.

First, the playful nature of the activities consistently elicited curiosity, interest, and active participation among all children, including those with special educational needs (whose data were not included in the analysis, but who participated in the sessions). A particularly relevant finding concerns the strong interest in replicability observed across participants, regardless of age. This aspect is noteworthy, as it suggests the potential use of the game not only as a screening tool but also as a support for intervention and rehabilitation pathways.

At the same time, several critical issues emerged, related to both technical and design factors. From a technical perspective, device performance occasionally affected the gameplay experience: limited processing speed led to slowdowns and malfunctions, including inconsistencies in the functioning of the memory-like mini-game.

From a design perspective, some mechanics proved less effective than expected. In the snake-like mini-game, for example, both the movement speed of the vehicle and the spatial arrangement of target objects raised usability concerns. These findings suggest that the difficulty level of Game 2 may have exceeded the capabilities of part of the target age group. Future versions of the game will therefore include a recalibration of task difficulty, movement dynamics, and object distribution to ensure a more age-appropriate assessment and greater sensitivity to individual differences in performance.

In addition, the control system – based on discrete 90-degree movements and lateral inputs – was not always intuitive, particularly for younger children, resulting in repeated attempts and, in some cases, frustration. A more fluid touch-based interaction (e.g., swipe gestures) could improve usability.

The “postman” mini game, although generally well received, also showed some limitations related to the complexity of the interaction mechanics, which tended to slow down task progression. Similarly, the reward system associated with village construction appeared unclear, as new elements were not always directly linked to player actions, reducing the perceived contingency between performance and feedback.

Further observations concern the relatively high number of dialogues. While this support narrative engagement, they may also slow down the overall pacing of the experience. Additionally, the need to expand the variety of stimuli (e.g., symbols, sequences, non-words) emerged, to reduce repetitiveness and sustain long-term motivation and replay-ability.

An additional limitation concerns the composition of the sample. Participants were recruited from a single primary school, which may limit the generalizability of the findings to broader populations. Future validation studies should therefore involve larger and more heterogeneous samples, including children from different educational, socio-economic, and geographical contexts.

Overall, these qualitative findings provide useful directions for system refinement, highlighting the importance of balancing usability, interaction variety, and feedback clarity to support both assessment effectiveness and the quality of the playful experience.

From a methodological perspective, future work should include testing with younger children (e.g., 5-year-olds) to verify the system’s effectiveness across the full target age range for dyslexia screening. Furthermore, expanding the game environments to incorporate phonological elements would allow the collection of data on an additional set of key pre-literacy skills.

The study did not control for previous gaming experience, familiarity with touchscreen devices, or game order effects. Future studies should include these variables and adopt randomized administration procedures.

Furthermore, expanding the game environments to incorporate phonological elements would allow the collection of data on an additional set of key pre-literacy skills.

## 5 Conclusion

Considering the findings, applied games should not be understood as a simple functional extension of play into educational or diagnostic contexts. Rather, they represent a hybrid and interdisciplinary field, bringing together perspectives from psychology, game design, digital technologies, and cultural studies. Within this framework, the value of games lies not in their instrumentalization, but in their capacity to preserve – and even enhance – their intrinsic playful dimension, leveraging it to support learning processes, promote well-being, and foster individual and social change.

Playfulness, conceived as a pedagogical tool, contributes to enhancing the learning environment (Brandwein, 2017) by incorporating elements of enjoyment and positive engagement, thereby fostering students’ capacity to remain motivated and comfortable when facing increasingly complex situations (Yanqui et al., 2017). Non-educational games, despite their name, have been frequently used for educational purposes (Squire, 2003; Wastiau et al., 2009; Martinez et al., 2022), such as teaching languages (Chen and Yang, 2013; Reinders, 2012), history (Squire, 2005), and science (de Aldama and Pozo, 2020). The findings further indicated that learning does not simply emerge as an incidental outcome of gameplay; rather, it is deeply intertwined with broader processes of motivational development. Consequently, although learning derived from non-educational gaming activities may appear trivial or purely recreational, such engagement may in fact be driven by an underlying, self-determined eudaimonic motivation oriented toward personal growth and learning (Vahlo et al., 2023).

The case of Starlex aligns with this perspective, demonstrating how digital environments can be designed so that affective and experiential dimensions are not subordinated to assessment, but instead become the context through which meaningful data emerge. The integration of emotional engagement and assessment goals represents a key challenge for the field of serious and applied games, requiring a careful balance between methodological rigor, user experience quality, and ethical awareness.

As a pilot study, the present findings should be interpreted as preliminary, intended to guide iterative refinement rather than provide definitive psychometric validation. However, the results suggest a promising outlook, while also highlighting areas that require further development, particularly in relation to the calibration of game mechanics, content design, and the management of sensitive data. At the same time, ongoing advances in digital technologies—especially the increasing integration of artificial intelligence in game systems—open new opportunities. Recent evidence (Wermann et al., 2026), based on large-scale empirical studies, indicates that video games can provide effective socio-relational learning environments, supported by mechanisms such as visual scaffolding and reduced cognitive load compared to more traditional approaches.

In this context, Starlex represents an initial step toward the development of tools that integrate assessment and experience, combining scientific rigor with accessibility. Future work will focus both on refining the model and expanding its application contexts, with the aim of developing increasingly inclusive, adaptive, and learner-sensitive game-based ecosystems for education and early screening.

## Data Availability

The original contributions presented in the study are included in the article/Supplementary material, further inquiries can be directed to the corresponding author/s.
